# Unexpected timely fracture union in matrix metalloproteinase 9 deficient mice

**DOI:** 10.1371/journal.pone.0198088

**Published:** 2018-05-31

**Authors:** Masato Yuasa, Masanori Saito, Cesar Molina, Stephanie N. Moore-Lotridge, Michael A. Benvenuti, Nicholas A. Mignemi, Atsushi Okawa, Toshitaka Yoshii, Herbert S. Schwartz, Jeffry S. Nyman, Jonathan G. Schoenecker

**Affiliations:** 1 Department of Orthopaedics and Rehabilitation, Vanderbilt University Medical Center, Nashville, Tennessee, United States of America; 2 Department of Orthopaedic Surgery, Tokyo Medical and Dental University, Tokyo, Japan; 3 Department of Pharmacology, Vanderbilt University, Nashville, Tennessee, United States of America; 4 Department of Pathology, Microbiology, and Immunology, Vanderbilt University Medical Center, Nashville, Tennessee, United States of America; 5 Department of Biomedical Engineering, Vanderbilt University, Nashville, Tennessee, United States of America; 6 Center for Bone Biology, Vanderbilt University Medical Center, Nashville, Tennessee, United States of America; 7 Department of Veterans Affairs, Tennessee Valley Health Care System, Nashville, Tennessee, United States of America; 8 Department of Pediatrics, Vanderbilt University Medical Center, Nashville, Tennessee, United States of America; University of Rochester, UNITED STATES

## Abstract

Immediately following a fracture, a fibrin laden hematoma is formed to prevent bleeding and infection. Subsequently, the organized removal of fibrin, via the protease plasmin, is essential to permit fracture repair through angiogenesis and ossification. Yet, when plasmin activity is lost, the depletion of fibrin alone is insufficient to fully restore fracture repair, suggesting the existence of additional plasmin targets important for fracture repair. Previously, activated matrix metalloproteinase 9 (MMP-9) was demonstrated to function in fracture repair by promoting angiogenesis. Given that MMP-9 is a defined plasmin target, it was hypothesized that pro-MMP-9, following plasmin activation, promotes fracture repair. This hypothesis was tested in a fixed murine femur fracture model with serial assessment of fracture healing. Contrary to previous findings, a complete loss of MMP-9 failed to affect fracture healing and union through 28 days post injury. Therefore, these results demonstrated that MMP-9 is dispensable for timely fracture union and cartilage transition to bone in fixed femur fractures. Pro-MMP-9 is therefore not a significant target of plasmin in fracture repair and future studies assessing additional plasmin targets associated with angiogenesis are warranted.

## Introduction

Fracture of a bone presents four principle problems; bleeding, susceptibility to infection, bone avascularity, and biomechanical dysfunction [1]. The acute phase response is the injury response system that resolves these problems in a precise temporal sequence over the course of approximately six weeks divided into a survival phase and reparative phase [2]. The survival phase occurs immediately following fracture and includes activation of the coagulation and inflammatory cascades which together provide hemostasis and prevent infection [3]. Once hemostasis is achieved and infection has been avoided, the reparative phase is initiated prompting robust angiogenesis and osteogenesis which resolve bone avascularity and biomechanical dysfunction [2, 3].

Molecularly, during the survival phase of the acute phase response, the pro-polymer fibrinogen is converted to the crosslinked polymer fibrin which is essential to control bleeding and prevent infection by trapping bacteria and recruiting leukocytes [4]. Once perceived to promote the reparative phase of fracture, we recently determined that, if not removed, fibrin is a barrier to fracture angiogenesis, and therefore resolution of both bone avascularity and biomechanical dysfunction leading to non-union [3]. Thus, the serine protease plasmin, the principal protease that degrades fibrin during the transition from the survival phase to the reparative phase of fracture repair, plays an essential role in fracture repair [3].

Initially it was postulated that the singular role of plasmin in a fracture acute phase response was to remove fibrin at the transition of survival and repair. However, depletion of fibrin alone was not sufficient to fully restore the fracture reparative phase in plasminogen deficient mice, suggesting additional plasmin targets may be integral for proper fracture repair [3]. Given that both a deficiency of plasmin and matrix metalloproteinase 9 (MMP-9) have been reported to inhibit fracture repair resulting in delayed bone union [3, 5], and that plasmin activates pro-MMP-9 to active MMP-9 [6] we hypothesize that plasmin activated MMP-9 promotes fracture repair and bony union. To test this hypothesis, we employed our fixed murine femur fracture model to serially assess fracture healing.

## Materials and methods

### Animals

Pro-MMP-9 deficient mice on a C57BL/6J background (JAX stock #007084) [7] and wild-type littermates were bred and housed in the animal facility of Vanderbilt University with a 12-hour light/dark cycle where water and food was provided *ad libitum*. To avoid sex-related confounding effects on developmental bone growth and fracture repair, only male mice were used in this study. All mice were 8 weeks of age at the time of fracture. Sample size calculations for assessing fracture healing were determined previously [8].

### Fixed femur fracture model

An open femur fracture model previously validated by our lab [2] was performed when mice were 8 weeks of age. Following adequate anesthetization and analgesia to minimize suffering, a 10-12mm long, medial incision was performed to expose the mid-shaft of the femur. The femur was then fractured in a controlled manner by scoring the bone and inducing a clean, transverse break with a beaver blade. Finally, the femur was then stabilized with the retrograde placement of a 23-gauge intramedullary pin, and the incision was closed using nylon sutures. Fracture repair was followed radiographically weekly, and mice were sacrificed at varying time points (7, 10, 14, 21, or 28) to allow for histologic, angiographic, and microcomputed tomography (μCT) analysis as described below. All animal protocols were reviewed and approved by the Institutional Animal Care and Use Committee (IACUC) of Vanderbilt University Medical Center (M1600231-00). Humane endpoints were in place to euthanize any animal that was in pain, unable to eat or drink, experienced wound dehiscence or infection, or lost > 20% of its original body weight. Mice were monitored every 12 hours for the first three days and then weekly until the point of sacrifice. Throughout these experiments no animals died or necessitated sacrifice prior to their designated endpoint. All mice in this study were euthanized by carbon dioxide inhalation followed by cervical dislocation.

### Fracture model output measurements

#### X-ray

Radiographic imaging was performed longitudinally to assess fracture healing. Mice were placed in the prone position and imaged 4 seconds at 35kV with a Faxitron LX-60 (Tucson, AZ). Radiographic images were then individually quantified for fracture healing by three independent observers assessing the following 3 criteria: 1. Bone formation, 2. Bone union, and 3. Bone remodeling [3]. Each criterion was scored as either “0” which means “does not meet the criteria” (i.e. not present/visible on the radiograph) or “1” which indicated that the criteria was met and is present by radiographic analysis. Operational definitions for each criteria point are as follows: 1) *Bone Formation*; 0 point: no apparent hard tissue callus formation, 1 point: apparent hard tissue callus formation. Yellow arrow indicates apparent hard tissue callus in x-ray and histology (S1 Fig—Top Panel). 2) *Bone Union*; 0: an existence of no mineralization between hard tissue calluses, 1: continuity of mineralization. A red arrow head indicates apparent radiolucent zone (x-ray) (S1 Fig—Middle Panel). 3) *Bone Remodeling*; 0: no apparent shrinkage in the size of hard tissue callus compared to maximum size of hard tissue callus, 1: apparent shrinkage in the size of hard tissue callus compared to maximum size of hard tissue callus. Yellow arrows indicate dynamic change of hard tissue callus from larger callus to smaller callus in the same mouse (x-ray) (S1 Fig—Bottom Panel). This scoring criterion was employed for both the superior (area 1) and inferior sides (area 2) of the femur resulting in a maximum score of 6 and a minimum score of 0 per image (S1 Fig).

#### Angiography

Perfusion with microfil vascular contrast (MV-122 Flow Tech Inc., Carver, MA) was conducted as previously described [3]. Due to similarity between density of bone and microfil contrast, all fracture samples that were perfused were decalcified in 0.5 M EDTA, pH 8, prior to μCT imaging (microCT40, Scanco Medical AG, Bassersdorf, Switzerland). 3D vascular reconstructions were then merged onto previously obtained X-ray images to further depict vascular progression using Adobe Photoshop software (San Jose, CA) [3, 9].

#### Micro-computed tomography (μCT)

μCT images to visualize angiography were acquired as previously described [3]. Briefly, following specimen harvest, microfil filled femurs were imaged at 55-kVp, 145-uA, 200-ms integration, 500 projections per 360 degrees of rotation, with a 20 μm isotropic voxel size. The microfil compound was segmented from soft tissues using a threshold of 220 per thousand (or 450.7mgHA/cm^3^), a Gaussian noise filter of 0.2 with a support of 1. Quantification of fracture vascularity was assessed by vascular volume (mm^3^) and vessel thickness of 100 slices on either side of the fracture.

#### Histological analysis

Specimens removed for histological analysis were fixed in 10% neutral buffered formalin and decalcified in 0.5M EDTA (pH 8.0) for a minimum of 5 days. Tissues were then dehydrated in graded series of ethanol, cleared, embedded in paraffin, and sectioned sagittally at 6μm for subsequent staining. Safranin orange/fast green staining (Safranin-O) was performed following deparaffinization and rehydration. Briefly, slides were placed in freshly filtered working Wiegert’s hematoxylin for 10 minutes and immediately washed in running tap water for 10 minutes. Slides were then placed in 0.1% fast green solution for 5 minutes and rinsed quickly in 1% acetic acid for 10 seconds. Slides were then placed in a 0.1% safranin-O solution for 5 minutes and dehydrated prior to mounting with permount. Total callus size and soft tissue callus size was quantified as previously described [2].

### Statistical analysis

Analysis between MMP-9 deficient mice and wild-type littermates was conducted with a non-parametric t-test (Mann Whitney). Alpha = 0.05. Analysis was conducted in GraphPad V6 (La Jolla, CA).

## Results

### MMP-9 is not essential for timely fracture healing in an internally fixed femur fracture model

**Fig 1:**
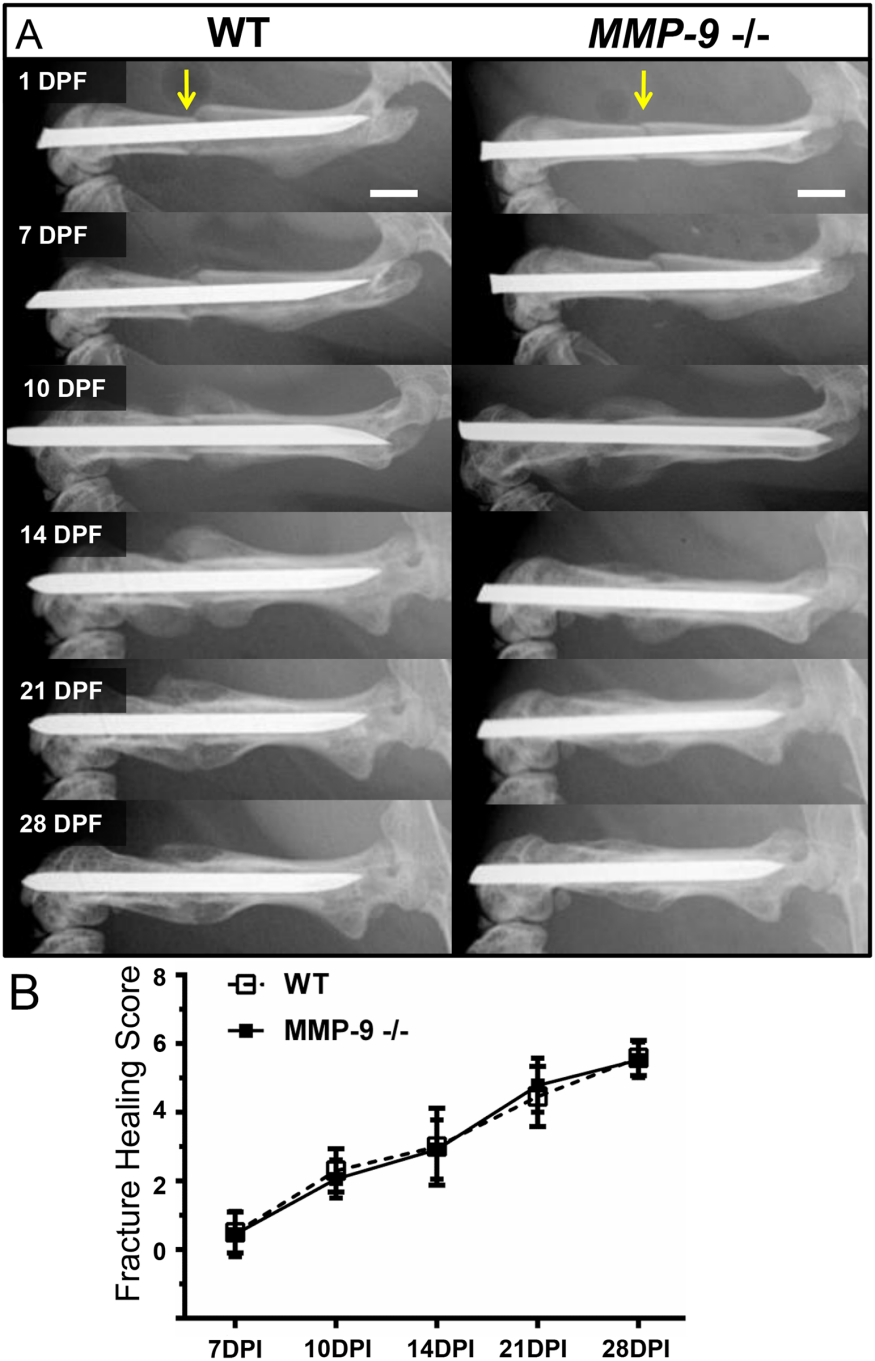
Skeletal healing of stabilized femur fracture in MMP-9 deficient mice. An open femur fracture model with stabilization by at 23-gauge intramedullary pin was used to compare key aspects of fracture healing in mice with and without MMP-9. To assess temporal development and subsequent remodeling of the hard tissue callus, we performed A) serial x-rays of the fractured femurs (yellow arrow indicates fracture site. Hard tissue callus is evident by X-ray proximal and distal to the fracture in mice with or without MMP-9 by 10 dpf. From 14 to 21 dpf, the proximal and distal hard tissue callus expands and coalesces at the fracture. At this point the hard tissue callus is at its maximum size and subsequently it starts remodeling through day 28. B) *Mmp-9 -/-* mice did not demonstrate any significant differences in fracture healing compared to WT littermates, based on scores of the bone formation, bone union, and bone remodeling. Data displayed represent the mean ± SD. Statistical significance between groups at each time point was determined using a non-parametric Mann-Whitney Test. Number of mice per genotype: 7dpf: WT- 22, MMP-9 KO- 35; 10d: WT-13, MMP-9 KO- 17; 14dpf: WT- 17, MMP-9 KO- 25; 21dpf: WT- 13, MMP-9 KO- 19; 28dpf: WT- 12, MMP-9 KO- 17.

Serial radiographs were performed at 1, 7, 10, 14, 21, and 28 days post fracture (dpf) to monitor development and remodeling of the hard tissue callus and subsequent fracture healing (S1 Fig). The hard tissue callus was clearly evident both proximal and distal to the fracture site in WT and *Mmp9-/-* mice starting at day 10 dpf and remodeled over the next two weeks (Fig 1A), resulting in comparable fracture union between groups at 14 dpf (Fig 1B). In comparison to WT littermates, mice lacking MMP-9 showed no qualitative or quantitative differences in the timing, growth, and remodeling of the hard tissue callus, or bone union. These results indicated that MMP-9 is not essential for fracture healing and contrary to previous reports [5], loss of MMP-9 does not lead to non-unions or delayed unions.

**Fig 2:**
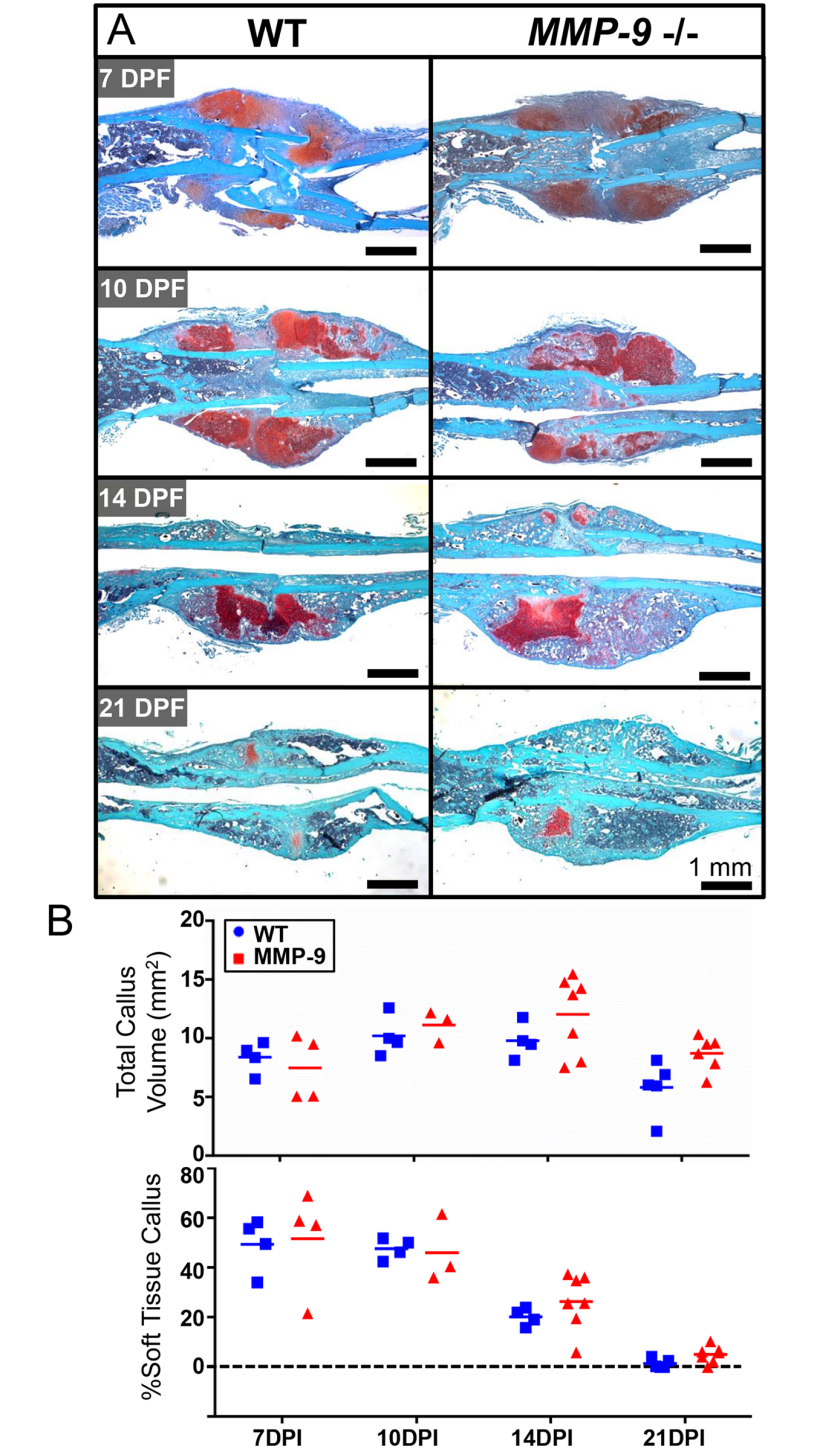
MMP-9 is not required for endochondral fracture healing. A) Both MMP-9 deficient and WT littermates possessed abundant soft cartilage at 7 and 10 dpf. At 21 dpf, hard tissue callus dominated in MMP-9 deficient and WT littermates. B) Quantification of the total callus volume and % soft tissue fracture callus demonstrated that there was no significant difference between MMP-9 deficient mice and WT littermates at any time assessed. While the % soft tissue callus area appears to trend higher in MMP-9 deficient mice at 14 DPI as previously described [5], there is marked variability between MMP-9 deficient mice.

To verify complete transition from early soft tissue callus, predominated by chondrocytes, into hard tissue callus, predominated by osteoblasts, osteoclasts, and endothelial cells, we prepared histological sections and stained with Safranin-O. Histologic evaluation of the fracture callus at 7, 10, 14, and 21 dpf revealed a central avascular chondroid matrix in both MMP-9 deficient and WT littermates at both 7 and 10 dpf, which then remodeled to hard callus by 21 dpf (Fig 2A and S2 Fig). Consistent with previous radiographic results, quantitative assessment of the soft tissue callus and total callus volume showed no statistical difference between WT and *Mmp-9 -/-* mice at any time point assessed (Fig 2B).

**Fig 3:**
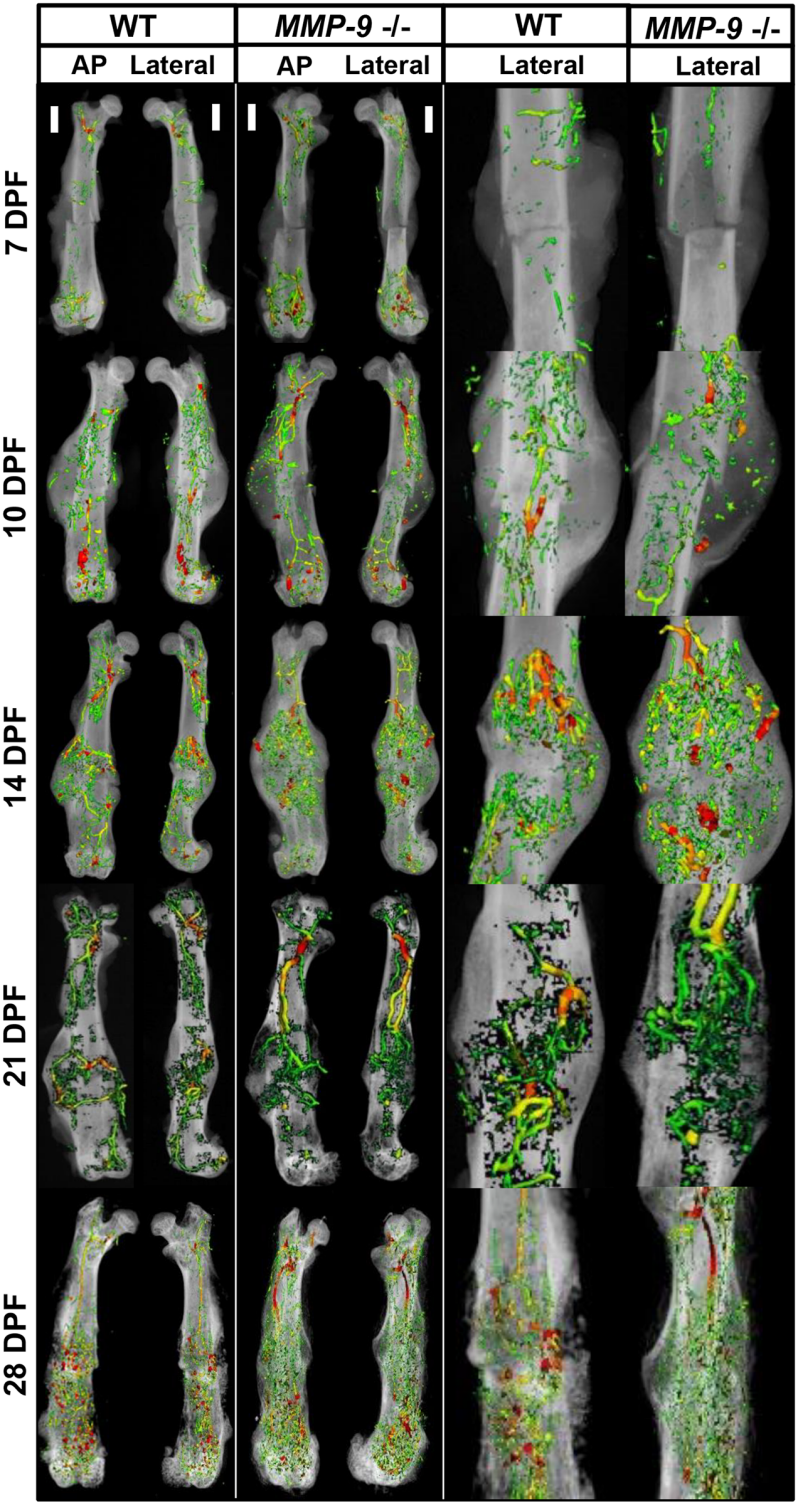
Revascularization of the fracture is unaffected by a loss of MMP-9. Visual representation of microfil based angiography overlaid with radiographic images. No differences in vascularity were observed between wild type and MMP-9 deficient mice at any time point.

**Fig 4:**
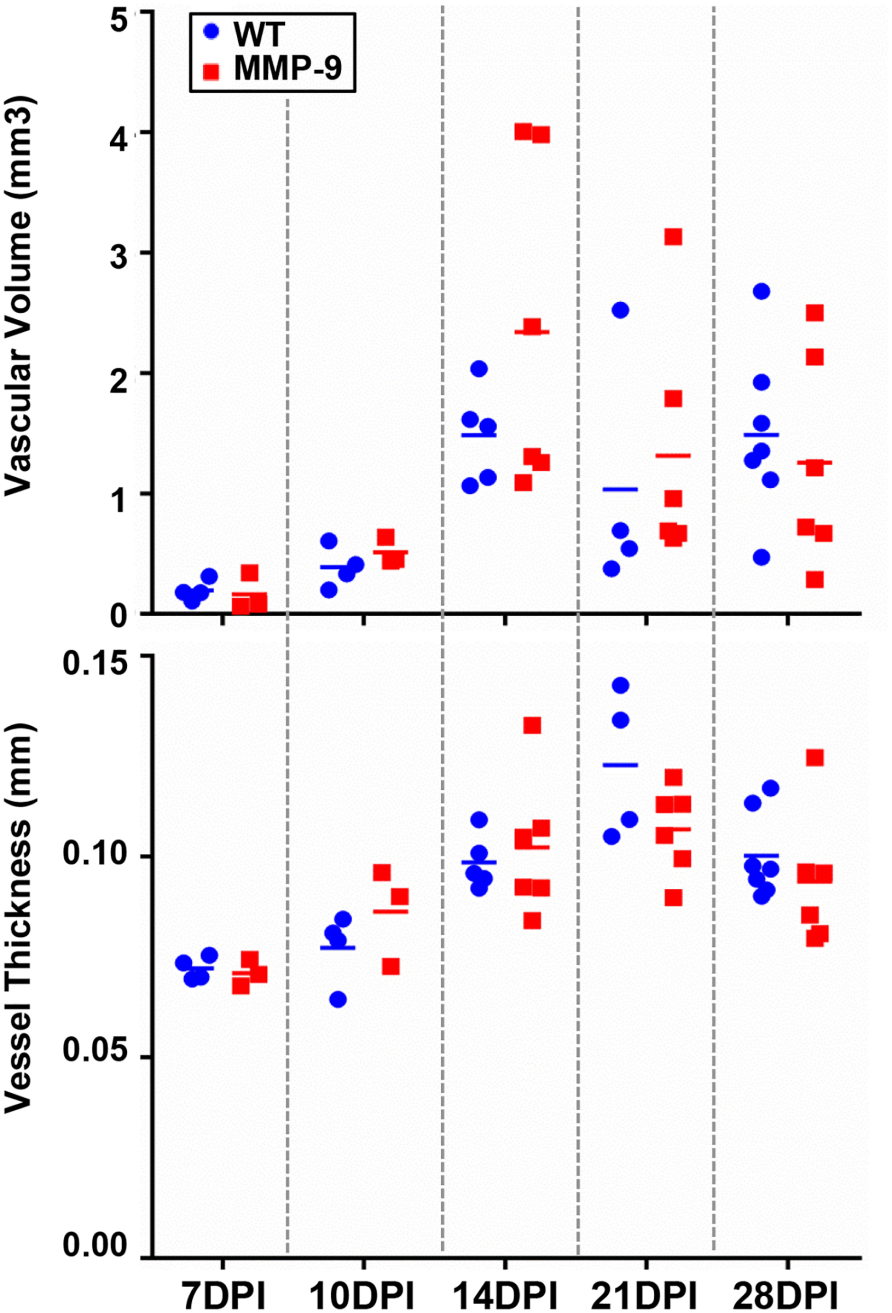
Vascular quantification of healing fractures of WT and MMP-9 deficient mice. Quantitative assessment of microfil based angiography surrounding the healing fracture. No statistical differences in vascularity were identified between wild type and MMP-9 deficient mice at any time point for either vascular volume (mm^3^) or vessel thickness (mm). 7d- WT: N = 4, MMP-9: N = 3; 10d- WT: N = 4, MMP-9: N = 3; 14d- WT: N = 5, MMP-9: N = 6; 21d- WT: N = 4, MMP-9: N = 6; 28d- WT: N = 7, MMP-9: N = 6. Individual time points were assessed by non-parametric t-tests. Alpha = 0.05.

To further assess fracture repair, we comprehensively assess the vascularity throughout the fracture callus, given that clinically, fracture revascularization is essential for bony union. X-rays of the fractured femurs merged with 3D μCT reconstructions of microfil perfused femurs identified a loss of vascularity in both wild-type and MMP-9 deficient mice at 7 dpf. However, by day 21 dpf, dense vascularity bridging the fracture site is observed in both groups. The vascularity then continued to coalesce and remodel through 28 dpf, such that no visible difference was observed between genotype (Fig 3). Through quantitative assessment, we were unable to detect differences in vascular volume or vessel thickness between wild-type and MMP-9 deficient mice (Fig 4).

## Discussion

We conclude that a deficiency of MMP-9 is does not result in a delay or failure of angiogenesis or bone union during fracture repair. Specifically, the loss of MMP-9 resulted in no deficiencies of secondary fracture repair (intramembranous or endochondral ossification), including the development of the soft tissue callus, subsequent angiogenesis, or the formation and union of the hard tissue callus in an internally stabilized femur fracture model. Therefore, pro-MMP-9 can be eliminated as a potential plasmin target affecting timely fracture healing.

Additionally, while these results appear dichotomous to the conclusion of the previous report [5], “that Mmp9-/- mice have non-unions and delayed unions of their fractures caused by persistent cartilage at the injury site” we would like to note that Colnot *et*. *al*. results noted that “by 19 days post fracture, the maximum force at failure was equivalent in WT and MMP-9 deficient calluses, demonstrating that the MMP-9 defect around the onset of bone remodeling was resolved” [5]. This data aligns with our findings that MMP-9 deficient mice underwent effective fracture healing with appropriate bony union, contrary to the conclusions previously drawn.

While assessment of the data alone suggests similar findings in regards to bone union, the previous report did observe a difference in cartilage remodeling in an unfixed tibial fracture model and ‘pathologic’ endochondral ossification in a fixed tibial fracture model. In this regard, we would like to note three main possible differences between these present findings and previous reports: 1) technical differences in the mode of fracture stabilization or 2) difference in the vascularity of the femur as opposed to the tibia, or 3) differences in the age/growth phase of the experimental animals.

It has been well described that interfragmentary strain, and therefore fracture stabilization, affects the type (primary or secondary) and timing of fracture repair [10–13]. Likewise, delaying stabilization during the early phases of fracture healing promotes secondary fracture repair characterized by a greater amount of endochondral ossification than strictly primary repair [14]. The great majority of the results from the previous report were derived from an un-fixed tibial fracture model [5, 15], whereas we employed internal fixation of femur fractures with a 23-gauge needle. As such, the differences in the timing of remodeling of the cartilage intermediate may simply be a consequence of the two different models. Along these lines, in the previous report, finding in the fixed tibial fracture model indicated ‘pathologic’ endochondral ossification in the setting of an MMP-9 deficiency as compared to the absence of endochondral ossification in wild type mice. However, it is well recognized that, although stabilization reduces the interfragmentary strain, due to both residual interfragmentary strain and hypoxic segments, the pin stabilization tibial fracture model does repair through secondary fracture repair the includes both intramembranous and endochondral ossification [16].

Secondly, these studies differ in location of the fracture with previous reports assessing the tibia and our reports assessing the femur. While both long bones, these locations differ by the rich vascular supply of the femoral shaft when compared to the watershed areas with low blood flow seen in the tibia; thereby suggesting that MMP-9 may perform different functions in a fracture with poor vascular supply [17, 18]. In addition to a change in anatomical fracture location, the mechanism by which the fracture was induced was different across studies. The current study utilizes an open osteotomy model, whereas the prior study employed a closed 3 point bending fracture jig method [5]. The closed fracture jig likely led to more soft tissue trauma and potentially further decreased perfusion of the tibial fractures. Additionally, given that most diaphyseal fractures are treated with intramedullary nailing resulting in endochondral ossification, we believe that internal needle fixation best represents current clinical practice [19, 20].

Finally, in this study, we utilized 8-week-old, male mice which, though sexually mature, are still undergoing appendicular and axial skeletal growth at the time of fracture repair. Prior studies utilized mice that were 12–20 weeks old and therefore skeletally mature. Previously, Lu *et*. *al*. has demonstrated that *Mmp-9* expression and angiogenic capacity of a healing tibial fracture is elevated in skeletally immature (juvenile) mice as compared to skeletally mature (adult) counterparts [21]. These findings would suggest that genetic loss of MMP-9 may impact fracture healing of skeletally immature mice to a greater extent than their adult counterparts. Therefore, this present study utilizing 8-week-old animals was designed to detect the greatest changes in fracture healing, yet no differences between groups was identified.

Though these present studies were aimed at assessing pro-MMP-9 as a target of plasmin essential for fracture healing, when applied to our stabilized femur fracture model. We did not detect any difference in fracture healing or bony union as previous reported. Therefore, pro-MMP-9 can be eliminated as a potential plasmin target affecting timely fracture healing. Future studies assessing additional plasmin targets associated with angiogenesis, such as VEGF-A, are warranted [22].

## Supporting information

S1 Fig Radiographic quantification of fracture healing. Fracture repair was quantified by three criteria: 1) bone formation, 2) bone union, and 3) bone remodeling. The anterior (area 1) and posterior (area 2) sides of the fracture callus were quantified individually a total score per femur was reported. Meets criteria (Yes) = score of 1. Does not meet criteria = score of 0. Maximum score per femur = 6. All radiographic images were assessed in a blinded manner by 3 individual observers from 1 to 4 weeks after fracture.
(TIF)

S2 Fig Sample histology of WT and MMP-9 deficient mice following fracture injury. Histological samples from representative fractured femurs of WT and MMP-9 deficient mice at 7, 10, 14, and 21 dpf. Sections 1–5 represent slices 200uM apart, beginning with the first full slice with callus and ending with a medial slide identified by the pin space. At 7 dpf we observed abundant soft tissue callus that gradually bridged and was replaced by hard callus from day 10 to 21 dpf. We observed no statistical difference in soft tissue callus percentage between WT and MMP-9 deficient mice at any time point (Fig 2).
(TIF)
