# Supplementary figures and images for: Unexpected timely fracture union in matrix metalloproteinase 9 deficient mice
Source: PLoS One. 2018 May 31;13(5):e0198088. doi: 10.1371/journal.pone.0198088 (PMC5978876; doi:10.1371/journal.pone.0198088)

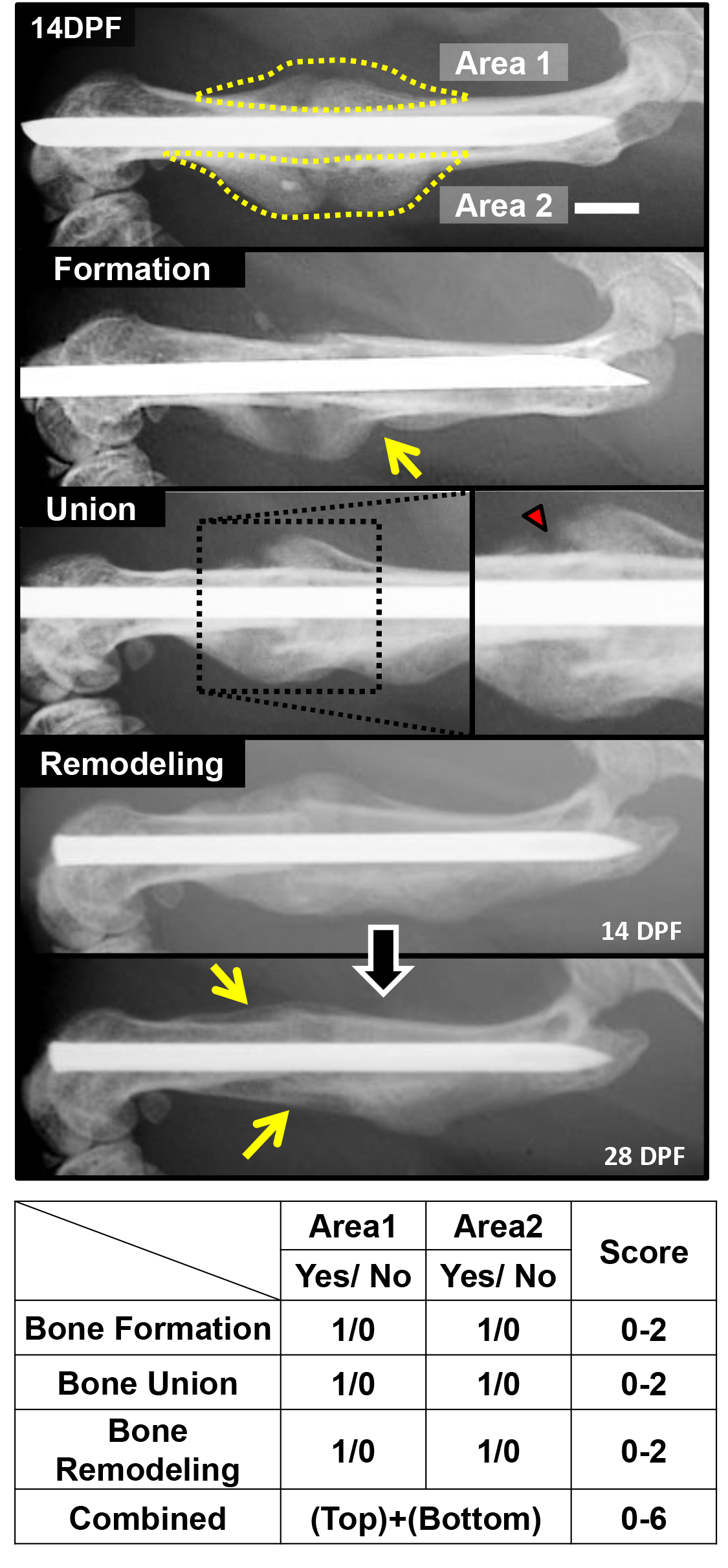

Supplement: S1 Fig — Fracture repair was quantified by three criteria: 1) bone formation, 2) bone union, and 3) bone remodeling. The anterior (area 1) and posterior (area 2) sides of the fracture callus were quantified individually a total score per femur was reported. Meets criteria (Yes) = score of 1. Does not meet criteria = score of 0. Maximum score per femur = 6. All radiographic images were assessed in a blinded manner by 3 individual observers from 1 to 4 weeks after fracture. (TIF) [file pone.0198088.s001.tif]

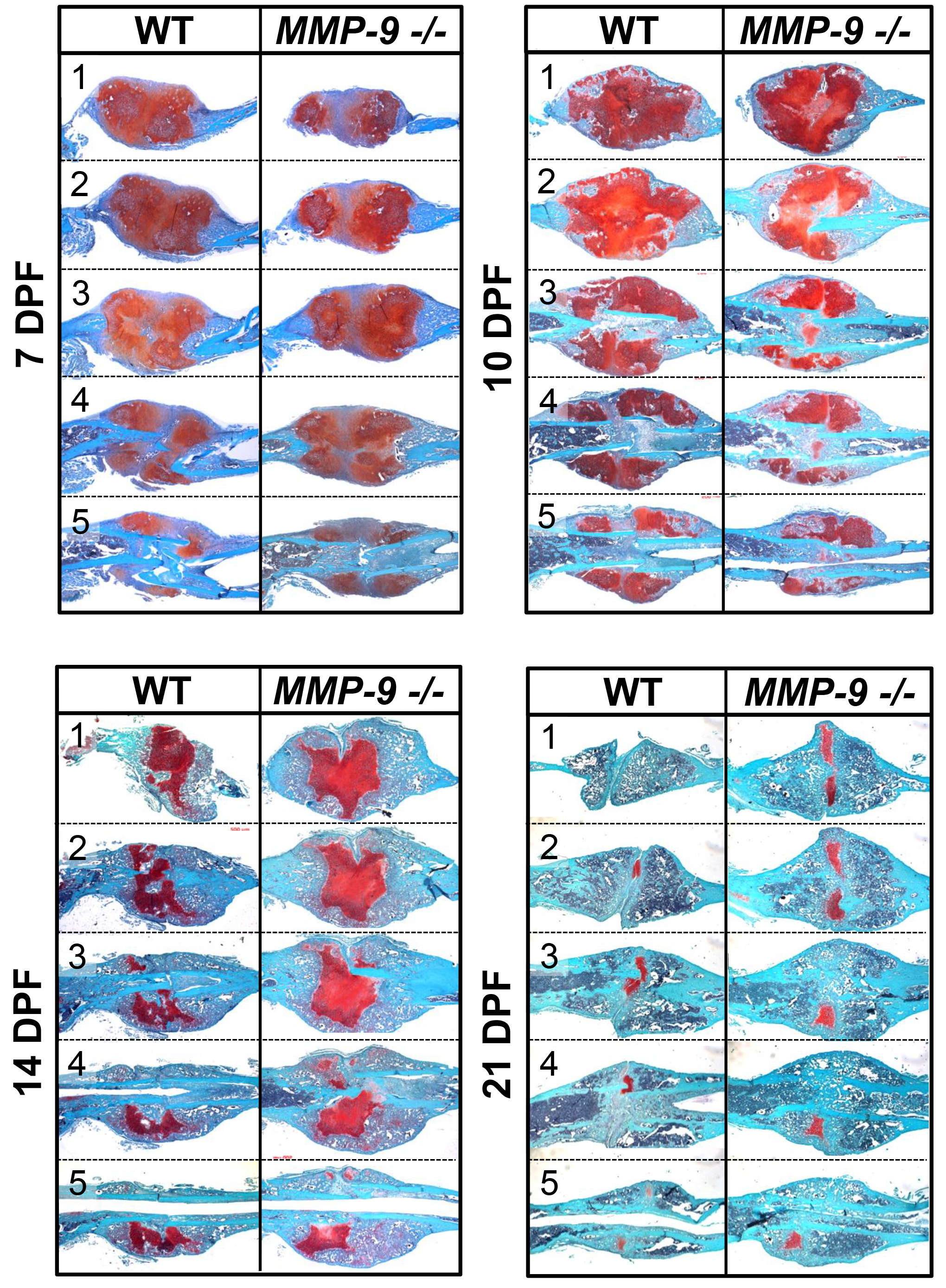

Supplement: S2 Fig — Histological samples from representative fractured femurs of WT and MMP-9 deficient mice at 7, 10, 14, and 21 dpf. Sections 1–5 represent slices 200uM apart, beginning with the first full slice with callus and ending with a medial slide identified by the pin space. At 7 dpf we observed abundant soft tissue callus that gradually bridged and was replaced by hard callus from day 10 to 21 dpf. We observed no statistical difference in soft tissue callus percentage between WT and MMP-9 deficient mice at any time point (Fig 2). (TIF) [file pone.0198088.s002.tif]
